# Prognostic value of microRNA-4521 in non-small cell lung cancer and its regulatory effect on tumor progression

**DOI:** 10.1515/med-2021-0312

**Published:** 2021-08-11

**Authors:** Butong Sun, Dan Cong, Kang Chen, Yuansong Bai, Jun Li

**Affiliations:** Department of Hematology and Oncology, China-Japan Union Hospital of Jilin University, Changchun, Jilin, 130031, China; Department of Hematology and Oncology, China-Japan Union Hospital of Jilin University, No. 126 of Xiantai Street, Changchun, Jilin, 130031, China

**Keywords:** NSCLC, miR-4521, biomarker, proliferation, migration, invasion

## Abstract

**Background:**

Non-small cell lung cancer (NSCLC) is a malignant tumor with the highest mortality rate in our country. It has been found in many studies that microRNA-4521 (miR-4521) is abnormally expressed and plays a role in clear cell renal cell carcinoma and other cancers.

**Objective:**

The purpose of this study was to explore the relationship between miR-4521 expression and clinical prognosis, as well as its influence on cell biological behavior.

**Methods:**

The expression differences of miR-4521 in NSCLC tissues and cells were examined by qRT-PCR technology. Kaplan–Meier survival analysis and Cox regression analysis were used to analyze the clinical information and survival status of patients to explore the relationship. Using the vitro cell MTT assay, Transwell assay, and western-blot analysis, the effects of miR-4521 on cell proliferation, migration, and invasion were analyzed.

**Results:**

The expression of miR-4521 in NSCLC tissues and cells was significantly downregulated. miR-4521 can be used as an independent prognostic factor. The survival rate of the miR-4521 low expression group was lower, which was significantly related to poor prognosis. In addition, the low expression of miR-4521 significantly promoted cell proliferation, migration, and invasion with highly expressed related protein levels. FOXM1 might be a direct target of miR-4521.

**Conclusion:**

The results of this study showed that the low expression of miR-4521 indicated the poor prognosis of NSCLC and promoted cell proliferation, migration, and invasion by targeting FOXM1.

## 1 Introduction

Lung cancer is the fastest-increasing incidence rate and the major malignant tumor among death-related cancers worldwide [1]. Non-small cell lung cancer (NSCLC) accounts for about 80% of lung cancer, of which lung adenocarcinoma (LUAD) and lung squamous cell carcinoma (LUSC) are the most common subtypes [2,3]. It is difficult to detect NSCLC in the early stage and most patients are already in the advanced stages when they are discovered, with poor clinical prognosis and a low 5 year survival rate [4,5]. The diagnosis and treatment of NSCLC have made progress in the past two decades, with surgical resection and adjuvant treatments as the mainstay [6,7]. The guidelines for the treatment of NSCLC that stands today were established with more biomarker-driven and effective-matched targeted therapies [8]. Tissue- and blood-based biomarker testing is vital for determining the optimal treatment for NSCLC patients. However, due to its high recurrence rate and metastasis rate, the survival rate after treatment is still very low [9]. At present, in the postoperative monitoring of NSCLC, TNM (tumor-node-metastasis) comprehensive staging and common tumor markers are generally used as prognostic factors, but their accuracy and specificity are poor [8]. Therefore, there is an urgent need to find new targets and markers for the treatment and prognosis of NSCLC.

MicroRNAs (miRNAs) are small noncoding RNAs with a length of about 17–22 nucleotides [10]. miRNAs regulate the gene expression of target proteins through transcription and can also be combined with other miRNAs to regulate target genes [11,12]. Studies have shown that miRNAs are involved in basic cell activities such as cell proliferation, differentiation, and apoptosis [13,14]. Recent studies have found that miRNAs are closely related to the development of cancers and can be used as cancer oncogene or tumor suppressor genes according to different expressions [15,16,17]. Previous studies have shown that many miRNAs are involved in the development of NSCLC by regulating gene expression, including miR-760, miR-126-3p, and miR-940 [18,19,20]. microRNA-4521 (miR-4521) has been reported to play a critical role in human cancers. For example, miR-4521 acts as a tumor suppressor in clear cell renal cell carcinoma (ccRCC) and miR-4521 deficiency contributes to ccRCC clinical progression and renal cancer cell malignant properties [21]. Zhang et al. obtained the top 40 differential expression levels of miRNAs including miR-4521 by the microarray analysis of NSCLC tissues and adjacent tissues [22]. However, the mechanism of miR-4521 in the development of NSCLC needs further research.

In this study, we examined the abnormal expression of miR-4521 in NSCLC tissues and cells. Then, we analyzed the correlation of its expression with clinical characteristics and survival status to understand its clinical significance. We also further explored its role in cell activities through *in vitro* cell experiments. This study may provide some theoretical support for the development of prognostic biomarkers for NSCLC patients.

## 2 Materials and methods

### 2.1 Patients and tissue specimens

We selected 131 patients who underwent NSCLC surgical resection in China–Japan Union Hospital of Jilin University between January 2013 and December 2014. The patients did not receive any other adjuvant treatments before surgery. Their clinical conditions are shown in Table 1. We took each patient’s cancerous tissues and adjacent normal tissues that were verified by pathologists. Then, we froze them in liquid nitrogen quickly. We ensured that each patient signed a consent form before surgery, agreeing to use their organization in this study. Each patient was followed up by telephone for 5 years. This study has been approved by the ethics committee of China–Japan Union Hospital of Jilin University.

**Table 1:** Correlation between miR-4521 expression levels and clinical features in NSCLC patients

Parameters	Case No. (*n* = 131)	miR-4521 expression		*P*
		High (*n* = 61)	Low (*n* = 70)	
Age				0.622
<60	70	34	36	
≥60	61	27	34	
Gender				0.778
Male	79	36	43	
Female	52	25	27	
Tumor size				0.083
<4 cm	71	38	33	
≥4 cm	60	23	37	
Histological type				0.761
Squamous cell carcinoma	54	26	28	
Adenocarcinoma	77	35	42	
Lymph node metastasis				0.016
Absent	69	39	30	
Present	62	22	40	
TNM staging				0.011
I–II	77	43	34	
III–IV	54	18	36	

### 2.2 Cell culture

Human NSCLC cell lines (H460, HCC821, A549, and H1975) and human normal lung epithelial cell line BEAS-2B were purchased from the Cell Bank of the Chinese Academy of Sciences. The cells were cultured in RPMI-1640 medium (Invitrogen, Carlsbad, CA, USA) supplemented with 10% FBS (Invitrogen, Carlsbad, CA, USA) in a humid 37°C incubator containing 5% CO_2_.

### 2.3 Cell transfection

First, the cells were cultured in six-well plates. Then, we used Lipofectamine 2000 (Invitrogen; Thermo Fisher Scientific, Inc.) to transfect miR-4521 mimic, mimic negative control (NC), miR-4521 inhibitor, or inhibitor NC (GenePharma, Shanghai, China) into the cells. The untreated cells were used as a blank control. All cells were quantified by qRT-PCR technology after transfecting.

### 2.4 RNA isolation and quantitative real-time PCR

We used TRIzol reagent (TaKaRa, Otsu, Shiga, Japan) to extract RNA from tissues and cell lines, and then used NanoDrop to determine the concentration of the extract to quantify RNA. Next, we used miRNA first-strand cDNA synthesis kit (Applied Biosystems, Foster City, CA) to reverse transcribe miRNA. According to the manufacturer’s instructions, we performed qRT-PCR on the 7900 real-time PCR system (Applied Biosystems, Foster City, CA) and used SYBR Green (GenePharma, Shanghai, China) to detect the miR-4521 expression in the tissues and cell lines. U6 was selected as the endogenous control and the relative value of its expression level was calculated by the 2^−ΔΔ*C*t^ method.

### 2.5 Cell proliferation

In this study, an 3-(4,5-dimethylthiazol-2-yl)-2,5-diphenyltetrazolium bromide) tetrazolium (MTT) assay was used to verify the effect of miR-4521 on cell proliferation. The transfected cells (1 × 10^4^ cells/well) were cultured in a 96-well plate and an MTT reagent (Sigma-Aldrich, St. Louis, MO, USA) was added to each well at 0, 24, 48, and 72 h. After incubating for 4 h, the cell suspension was removed and dimethyl sulfoxide (Sigma-Aldrich, St. Louis, MO, USA) was added. Finally, the absorbance was measured at 490 nm using a microplate reader.

### 2.6 Cell migration and invasion assay

The cell migration and invasion assays were verified with the Transwell chamber (24-well plate, 8 μm pore size; Corning Costar, Lowell, MA, USA), and the invasion assay required Matrigel (BD Biosciences, Franklin Lakes, NJ, USA) pre-coated on the bottom of the upper chamber. We cultured the transfected cells in a serum-free medium for a certain period and then added the cell suspension (5 × 10^4^ cells/well) to the upper chamber of the Transwell. At the same time, the serum-containing medium as a chemokine in the lower chamber was also added. After culturing for 24 h at 37°C, the cells in the upper chamber were fixed in methanol for 30 min and then stained with 1% crystal violet for 15 min. Then, the number of cells were counted using a microscope.

### 2.7 Western blot analysis

A549 cell lysates were prepared with RIPA buffer (Santa Cruz Biotechnology) containing 1% phenylmethylsulfonyl fluoride on ice and then centrifuged at 12,000 rpm at 4°C. The supernatant was collected and the protein concentrations were measured using the bicinchoninic acid method (Thermo Scientific). Lysates with equal amounts of protein were used for electrophoresis using 10% SDS-PAGE for protein separation. Then, the protein was transferred onto the poly (vinylidene fluoride) membrane (Millipore, Bedford, USA) in Tris/glycine/SDS buffer and blocked in 5% milk for 1 h. The membranes were incubated with the following primary antibodies: anti-cyclin D1 antibody (ab16663; Abcam, USA), anti-Cdk4 antibody (ab108357; Abcam, USA), anti-MMP2 antibody (ab92536, Abcam, USA), anti-MMP9 antibody (ab76003, Abcam, USA), and anti-β-actin (ab8227, Abcam, USA) overnight. The blots were washed with TBST and were exposed to HRP-labeled goat anti-rabbit IgG secondary antibodies (ab7090, Abcam, USA) for 1 h. Protein bands were visualized by electrochemiluminescence (ECL) and scanned by the software ChemoiDox MP system (Bio-Rad, USA).

### 2.8 Bioinformatic analysis and dual-luciferase reporter assay

Bioinformatic analysis using TargetScan 7.2 database showed that FOXM1 possesses binding sites of miR-4521 with a high context score percentile of 98. FOXM1 with miR-4521-binding sites was inserted into the pGL3 plasmid to construct the FOXM1 wild-type (WT) plasmid. Besides, the mutant miR-4521-binding sequence was synthesized to construct the FOXM1 mutant (MUT) plasmid. A549 cells (with the lowest miR-4521 expression levels) were incubated in 48-well plates for 24 h. Then, cells were transfected with FOXM1-WT or FOXM1-MUT plasmid in combination with miR-4521 mimic, inhibitor, or controls using Lipofectamine 2000 (Invitrogen; Thermo Fisher Scientific, Inc.). After transfection for 48 h, a Dual-Luciferase Assay kit (Promega) was used to analyze the relative luciferase activities.

### 2.9 Statistical analysis

SPSS22.0 software (SPSS Corporation, Chicago, Illinois, USA) and GraphPad 7.0 (GraphPad, La Jolla, California, USA) were used for statistical analysis. The significant differences between different groups were analyzed by Student’s *t*-test or one-way ANOVA. Kaplan–Meier analysis and Cox regression analysis were used to determine the relationship between miR-4521 expression level and clinical characteristics of patients. All determinations were repeated more than three times, and the results are expressed as mean ± SD. *P* < 0.05 means significant.

## 3 Results

### 3.1 miR-4521 expression in NSCLC tissues and cell lines

**Figure 1:**
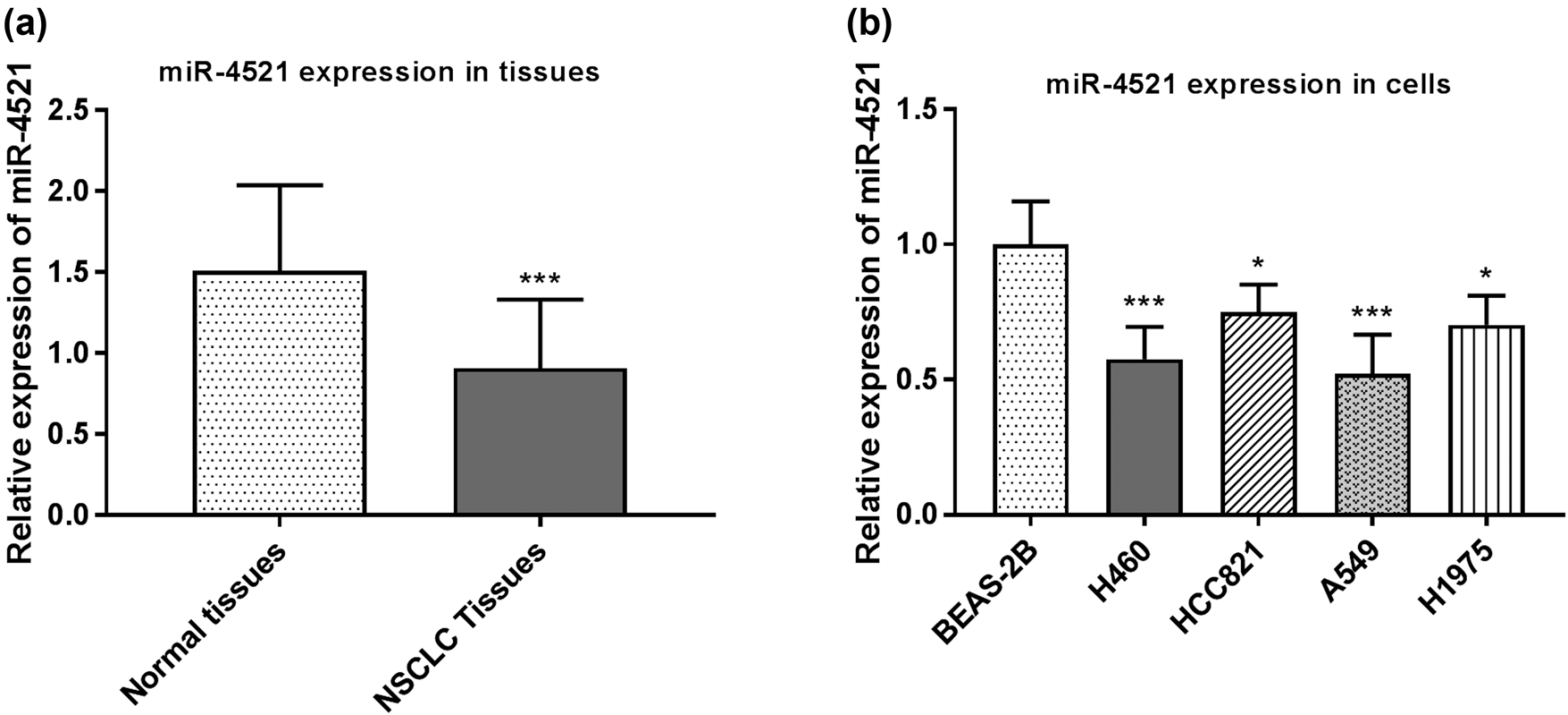
The relative expression of miR-4521 in NSCLC tissues and cells. (a) Compared with adjacent noncancerous lung tissues, the expression of miR-4521 in NSCLC tissues was significantly downregulated (***P < 0.001). (b) Compared with the lung epithelial cell BEAS-2B, the expression of miR-4521 in the NSCLC cell lines (H460, HCC821, A549, H1975) was significantly downregulated (*P < 0.05, ***P < 0.001).

We first examined the expression of miR-4521 in 131 pairs of NSCLC tissues and adjacent noncancerous tissues by qRT-PCR. It can be seen from the results that the expression of miR-4521 in NSCLC tissues was significantly lower than that in adjacent noncancerous tissues (*P* < 0.001, Figure 1a). Next, the expression difference of miR-4521 in NSCLC cell lines and lung epithelial cell lines was determined. Compared with the lung epithelial cells BEAS-2B, the expression of miR-4521 in NSCLC cell lines (H460, HCC821, A549, and H1975) was significantly downregulated (*P* < 0.001, Figure 1b). We selected two groups of cells A549 and H460 with lower relative expression from the four cell lines for the next experiments.

### 3.2 Expression of miR-4521 was correlated with clinicopathological features of NSCLC patients

We divided NSCLC patients into high-expression group (*n* = 61) and low-expression group (*n* = 70). The grouping was based on the average expression of miR-4521 in the tissues of NSCLC patients. Table 1 shows the correlation between the downregulation of miR-4521 and clinicopathological indicators. It can be concluded from the table that the low expression of miR-4521 is significantly correlated with lymph node metastasis (*P* = 0.016) and TNM staging (*P* = 0.011), but is not correlated with other parameters including age, gender, tumor size, and histological type (all *P* > 0.05).

### 3.3 miR-4521 was correlated with poor prognosis in NSCLC patients

**Figure 2:**
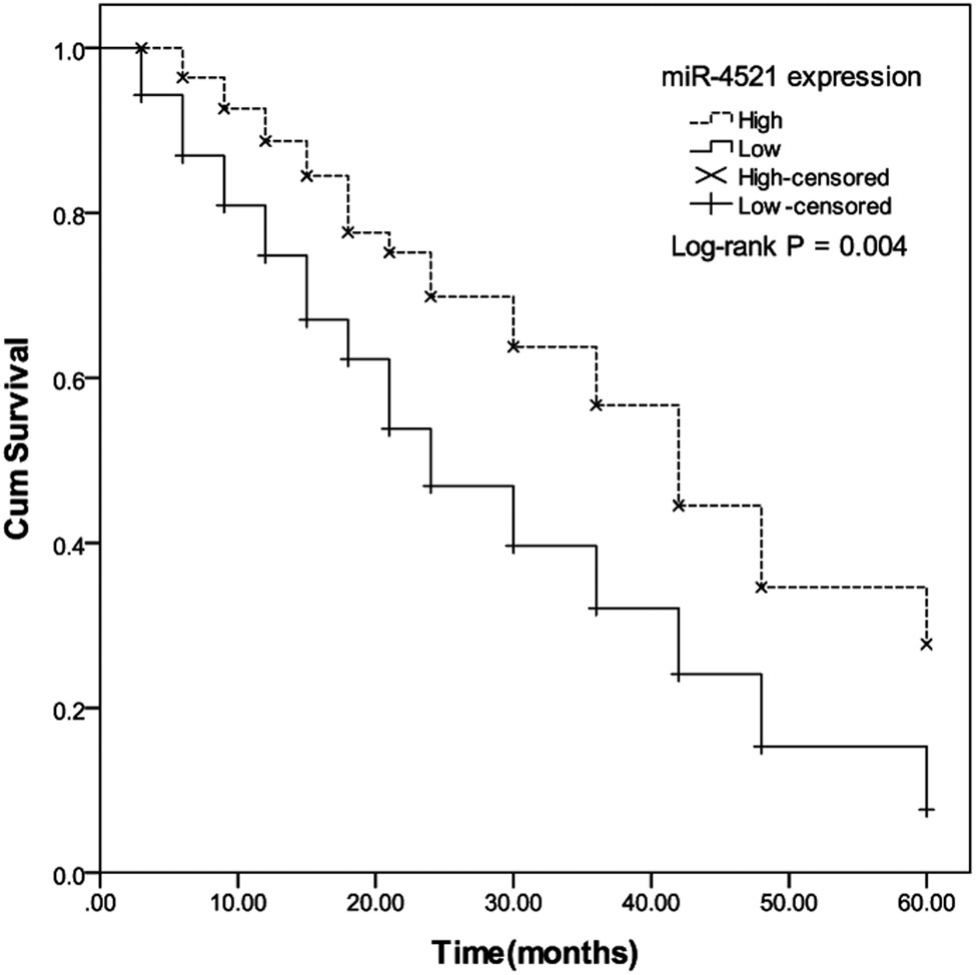
Survival curves of miR-4521 high expression group and low expression group. The 5 year survival rate of the miR-4521 high expression group was significantly higher than that of the low expression group (log-rank test *P* = 0.004).

The relationship between miR-4521 expression and prognosis was examined by survival curve and Cox regression model. It can be concluded from the Kaplan–Meier survival curve in Figure 2 that the 5 year survival rate of the high expression group was significantly higher than that of the low expression group, indicating that the low expression of miR-4521 may indicate the poor prognosis of NSCLC patients (log-rank test *P* = 0.004). The multivariate Cox risk regression model was used to analyze the relationship between the expression of miR-4521 in tissues and the survival status of the corresponding patients. It can be concluded that the expression of miR-4521 (HR = 0.544, 95% CI = 0.329–0.898, *P* = 0.017), lymph node metastasis (HR = 0.613, 95% CI = 0.379–0.991, *P* = 0.046), and TNM staging (HR = 0.575, 95% CI = 0.355–0.932, *P* = 0.017) can be used as independent factors for the prognosis of NSCLC (Table 2).

**Table 2:** Multivariate Cox analysis of clinical parameters in relation to overall survival

Characteristics	Multivariate analysis		
	HR	95% CI	*P*
miR-4521	0.544	0.329–0.898	0.017
Age	1.542	0.967–2.459	0.069
Gender	0.820	0.518–1.298	0.397
Tumor size (cm)	1.525	0.950–2.446	0.081
Histological type	0.737	0.449–1.211	0.229
Lymph node metastasis	0.613	0.379–0.991	0.046
TNM staging	0.575	0.355–0.932	0.025

HR: hazard ratio; CI: confidence interval.

### 3.4 Low expression of miR-4521 promoted proliferation, invasion, and migration of NSCLC cells

**Figure 3:**
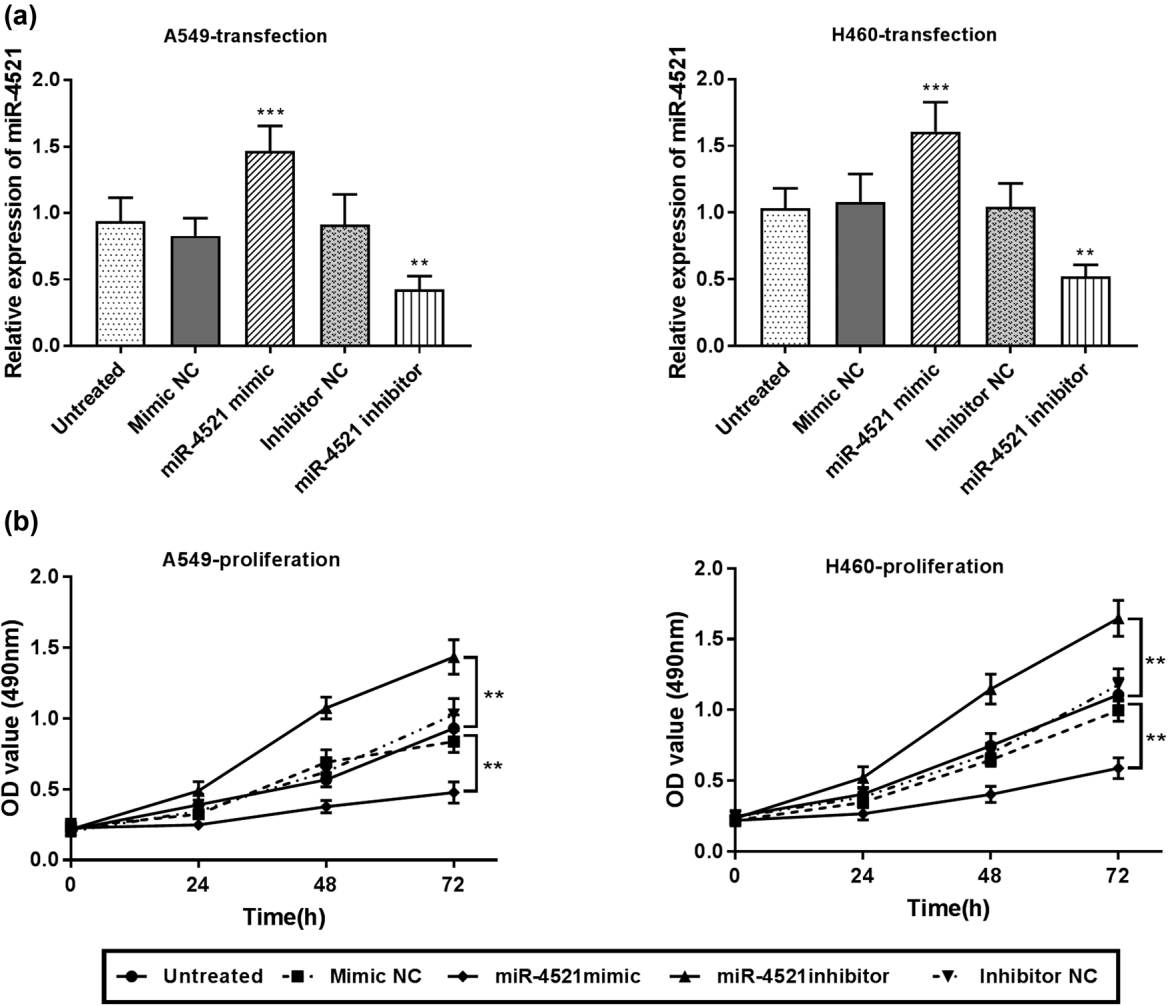
The effect of the miR-4521 expression on cell proliferation. (a) miR-4521 mimic promoted the expression of miR-4521, while miR-4521 inhibitor suppressed its expression (**P < 0.01, ***P < 0.001). (b) miR-4521 mimics can suppress A549 and H460 cell proliferation, while miR-4521 inhibitors can promote cell proliferation (**P < 0.01).

**Figure 4:**
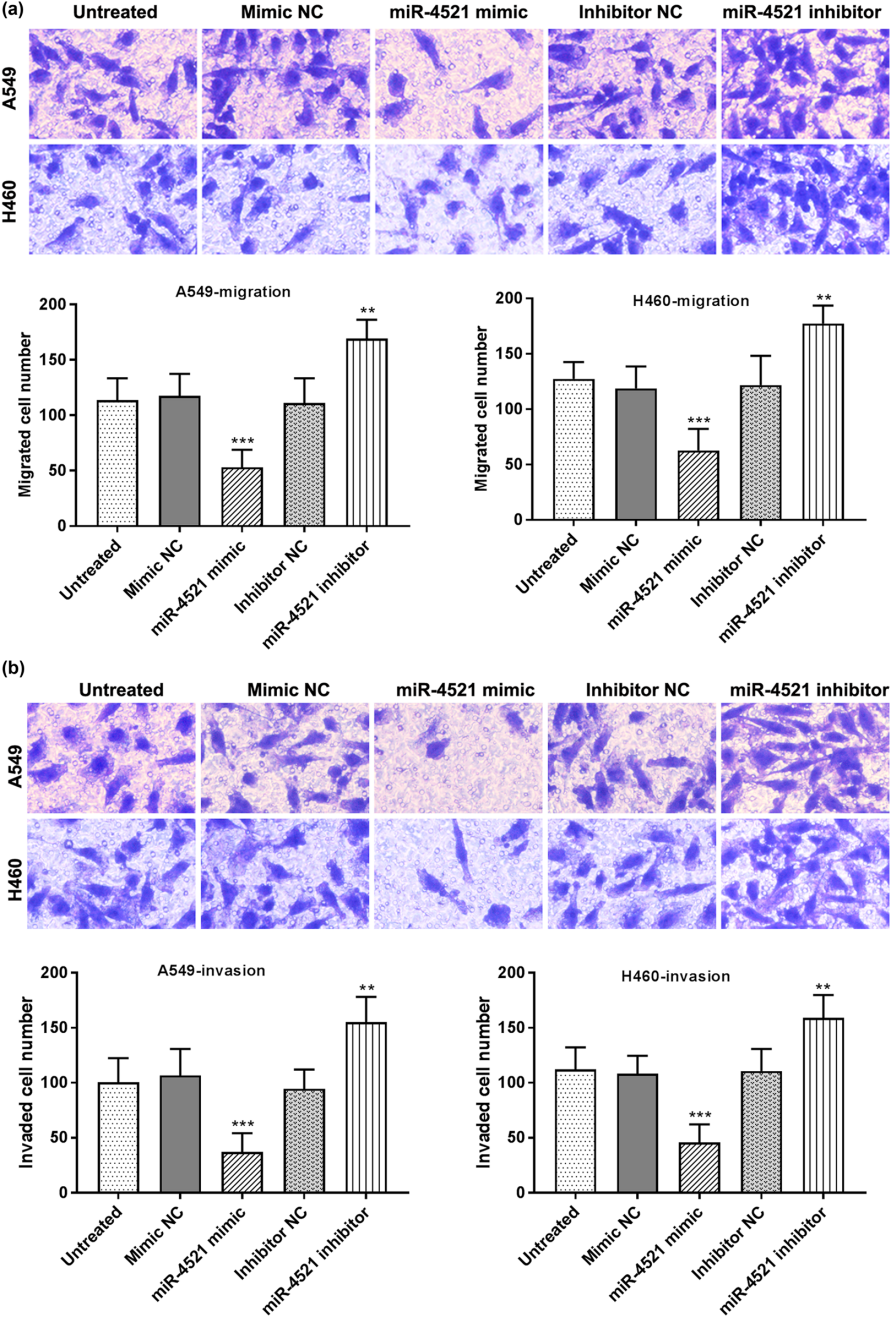
The effect of miR-4521 on cell migration and invasion. (a) miR-4521 mimics inhibited the migration of A549 and H460 cells, while miR-4521 inhibitors promoted cell migration (magnification ×200; **P < 0.01, ***P < 0.001). (b) miR-4521 mimics inhibited the invasion ability of cells, while miR-4521 enhanced the invasion ability of cells (magnification ×200; **P < 0.01, ***P < 0.001).

*In vitro* cell assays were conducted to explore the effects of miR-4521 on the proliferation, migration, and invasion of NSCLC cells by transfecting miR-4521 mimics or inhibitors into A549 and H460 cells. The detection of transfected cells by qRT-PCR revealed that miR-4521 inhibitors significantly downregulated the miR-4521 expression in A549 and H460 cells (*P* < 0.001), while the miR-4521 mimics significantly increased its expression in cells (*P* < 0.01) (Figure 3a). The transfected cells were analyzed by MTT assay to analyze the effect of miR-4521 on cell proliferation. It can be concluded that miR-4521 mimics significantly inhibited cell proliferation, while inhibitors significantly promoted cell proliferation (*P* < 0.01; Figure 3b). Next, we studied the effect of miR-4521 on the migration and invasion of A549 and H460 cells by the Transwell assay. It can be seen from the figure that miR-4521 mimics inhibited cell migration and invasion capabilities (*P* < 0.001), while miR-4521 inhibitors significantly enhanced cell migration and invasion capabilities (*P* < 0.01) (Figure 4a and b).

### 3.5 Effect of the miR-4521 expression on the corresponding cell proliferation and metastasis-related proteins

**Figure 5:**
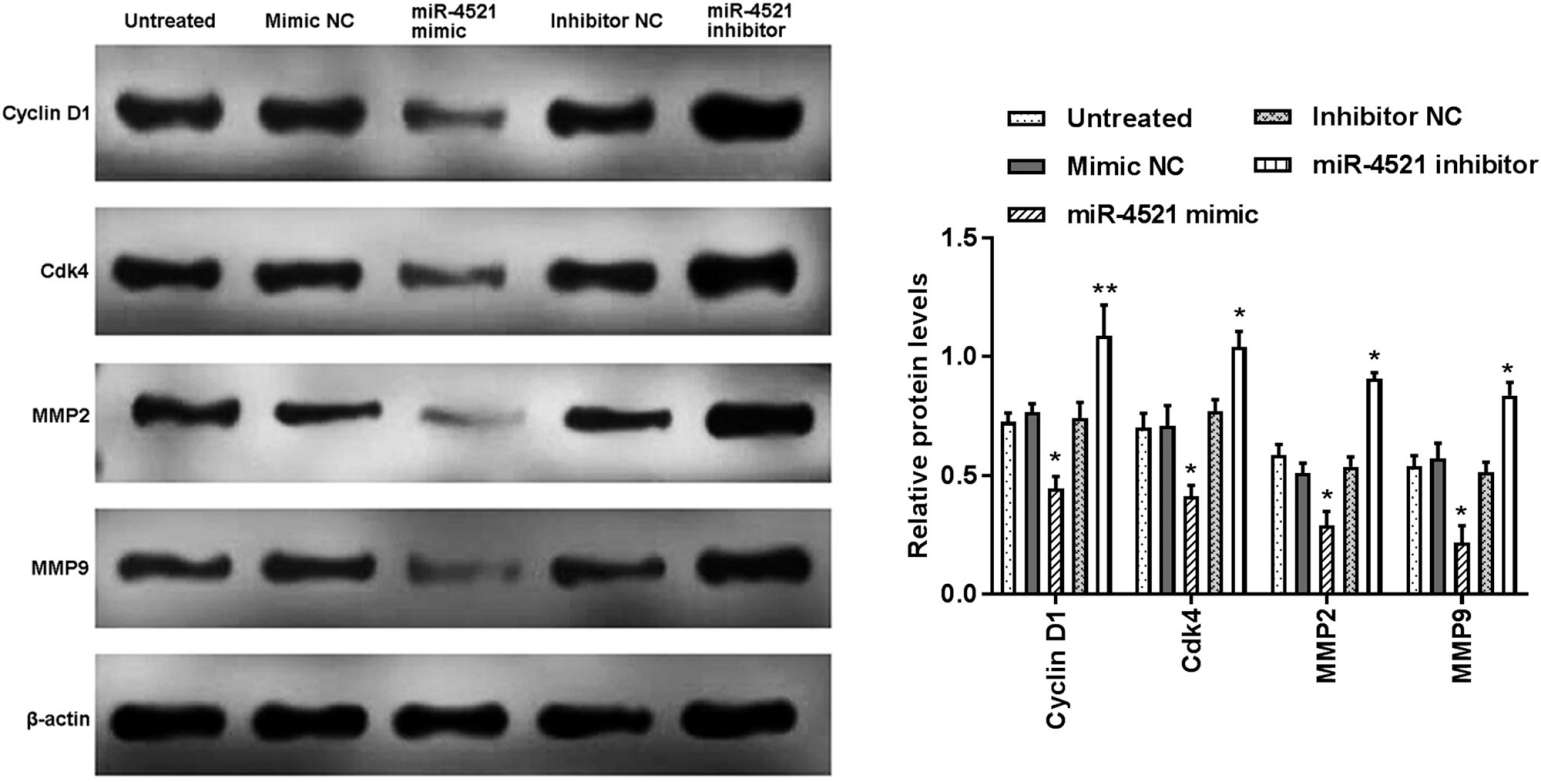
Cell proliferation-, migration-, and invasion-related protein levels were measured by western blot analysis. Enhanced miR-4521 expression inhibited the cyclin D1, Cdk4, MMP2, and MMP9 levels (*P < 0.05).

Cyclin D1, Cdk4, MMP2, and MMP9 are cell proliferation, migration, and invasion-related proteins. Thus, we further measured the expression of these proteins in A549 cells transfected with mimic, inhibitor, or NCs using Western blot assay. miR-4521 overexpression led to the downregulation of cyclin D1, Cdk4, MMP2, and MMP9 (Figure 5).

### 3.6 FOXM1 is directly regulated by miR-4521

**Figure 6:**
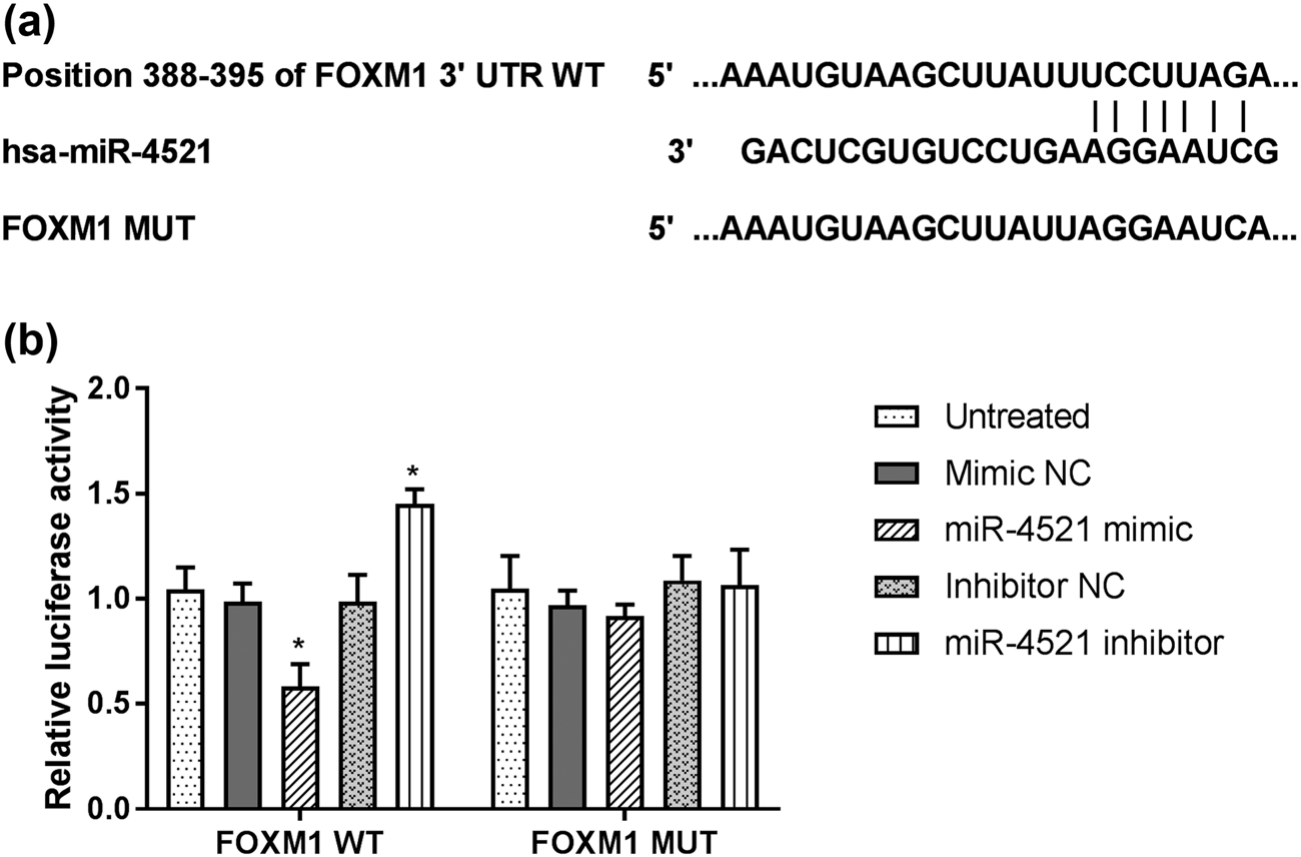
FOXM1 may be a direct target gene of miR-4521. (a) The binding sites between FOXM1 3′-UTR and miR-4521. (b) Dual-luciferase reporter assay was performed to confirm FOXM1 as a direct target of miR-4521 (*P < 0.05).

To uncover the potential mechanism of the decreased expression of miR-4521 in NSCLC cells, the potential targets of miR-4521 were identified using Targetscan prediction tools. Among these targets that were predicted, Forkhead Box M1 (FOXM1) was further studied. The binding sites between FOXM1 3′-UTR and miR-4521 are shown in Figure 6a. Subsequently, a dual-luciferase reporter assay was performed to confirm the hypothesis. As shown in Figure 6b, the luciferase activity of cells co-transfected with FOXM1 WT and miR-4521 mimics was significantly lower than that of the control group, while co-transfected with FOXM1 WT and inhibitors was higher than the control group (*P* < 0.05). However, the luciferase activities of cells co-transfected with FOXM1 MUT and mimics had no significant changes. Thus, we believed that FOXM1 is a downstream target of miR-4521 in NSCLC cells.

## 4 Discussion

NSCLC is the major pathological type of lung cancer, with high morbidity and mortality, which is easy to relapse and difficult to be monitored [23]. Therefore, in-depth study of the development mechanism of NSCLC and finding new biomarkers are the main research directions in the current field [24,25]. In recent studies, miRNAs have been found to be used as oncogenes or tumor suppressors and expressed differentially in various cancers [26,27]. Many studies explored the relevance of miRNAs to cancer prognosis and their effects on cell activities [28,29]. For example, Ma et al. have confirmed that the low expression of miR-100 was significantly associated with poor prognosis [30]. Feng et al. have proved that miR-16-1-3p may suppress the proliferation, migration, and invasion of NSCLC cells [31]. It was found that miR-4521 was abnormally expressed in cancers such as chronic lymphocytic leukemia (CLL) and had a certain impact on the migration, invasion, and apoptosis of ccRCC cells [21,32]. So, our study explored the abnormal expression and role of miR-4521 in the occurrence and development of NSCLC.

miR-4521 may be used as a tumor suppressor gene for NSCLC. Our study has found that compared with the control group, the expression of miR-4521 in NSCLC tissues and cell lines was significantly downregulated. By analyzing the relationship between the expression of miR-4521 and the clinical characteristics of patients, it can be concluded that the low expression of miR-4521 is significantly related to TNM staging and lymph node metastasis. These results are consistent with the existing research results. Pekarsky et al. have proved that miR-4521 was downregulated in tumor samples from patients with CLL [32]. Feng Xue et al. have demonstrated that miR-4521 deficiency promoted the progression of ccRCC in patients [21].

miR-4521 may be a prognostic biomarker of NSCLC. We used the Kaplan–Meier survival curve and Cox regression model to explore the relationship between miR-4521 expression and patient prognosis. It can be concluded from the survival curve that the survival rate of patients with low expression is lower and low expression of miR-4521 indicates a poor prognosis. At the same time, the Cox regression model can prove miR-4521 is an independent prognostic factor for NSCLC. Liao et al. have found that miR-4521 showed a good prognostic ability and can be used as an independent prognostic factor for the survival of pancreatic ductal adenocarcinoma [33]. These results are consistent with the results of our research and we can conclude that miR-4521 may be a marker of cancer prognosis.

The low expression of miR-4521 promoted the proliferation, migration, and invasion of NSCLC cells. We studied the effects of miR-4521 mimics or inhibitors on the biological behavior of NSCLC cells using MTT and Transwell assays. These results showed that low expression of miR-4521 can enhance cell proliferation, migration, and invasion ability, while high expression of miR-4521 can reduce its ability. Furthermore, enhanced expression of miR-4521 decreased the proliferation-, metastasis-related protein levels, including cyclin D1, Cdk4, MMP2, and MMP9. These data suggested that miR-4521 influences A549 cell malignancy via cyclin D1, Cdk4, MMP2, and MMP9. A previous study also demonstrated that the overexpression of miR-4521 can reduce the *in vitro* migration and invasion ability of ccRCC cells 786-O and ACHN through the TIMP-1/MMP2/MMP9 pathway, and promote their apoptosis through the MDM2/p53/Bcl2/Bax pathway to reduce its proliferation [21]. Senfter et al. have confirmed that miR-4521 transfection reduced the proliferation and invasion of several medulloblastoma cell lines by activating caspase 3/7, and induced programmed cell death [34]. Furthermore, we explored the potential downstream target of miR-4521 and FOXM1 might be a direct target of miR-4521. Mammalian transcription factor FOXM1, one of the members of the Forkhead family proteins, plays crucial roles in tumorigenesis in human cancers [35]. FOXM1 was reported to be upregulated in lung cancer (including NSCLC) and was associated with poor prognosis of patients, as well as regulating cell proliferation, apoptosis, and metastasis of cancer cells [36,37]. Based on these previous studies and present results, it is supposed that the abnormal expression of miR-4521 plays a certain role in the activities of NSCLC cells by targeting FOXM1. However, the detailed mechanism of miR-4521 in the development of NSCLC remains to be explored.

This study indicated that the expression of miR-4521 is downregulated in NSCLC tissues and cell lines, and its low expression is significantly related to the poor prognosis of patients. The low expression of miR-4521 promoted cell proliferation, migration, and invasion by targeting FOXM1. In short, miR-4521 may serve as a new prognostic biomarker and the miR-4521/FOXM1 axis may be a therapeutic target for the treatment of NSCLC patients.
